# Na_V_1.2 haploinsufficiency in *Scn2a* knock-out mice causes an autistic-like phenotype attenuated with age

**DOI:** 10.1038/s41598-019-49392-7

**Published:** 2019-09-09

**Authors:** Isabelle Léna, Massimo Mantegazza

**Affiliations:** 1 Université Côte d’Azur, 660 Route des Lucioles, 06560 Valbonne - Sophia Antipolis, France; 2 CNRS UMR 7275, Institute of Molecular and Cellular Pharmacology (IPMC), 660 Route des Lucioles, 06560 Valbonne - Sophia Antipolis, France; 3 Inserm, 660 Route des Lucioles, 06560 Valbonne - Sophia Antipolis, France

**Keywords:** Ion channels in the nervous system, Epilepsy, Autism spectrum disorders, Channelopathies

## Abstract

Mutations of the *SCN2A* gene, encoding the voltage gated sodium channel Na_V_1.2, have been associated to a wide spectrum of epileptic disorders ranging from benign familial neonatal-infantile seizures to early onset epileptic encephalopathies such as Ohtahara syndrome. These phenotypes may be caused by either gain-of-function or loss-of-function mutations. More recently, loss-of-function *SCN2A* mutations have also been identified in patients with autism spectrum disorder (ASD) without overt epileptic phenotypes. Heterozygous *Scn2a* knock-out mice (*Scn2a*^+/−^) may be a model of this phenotype. Because ASD develops in childhood, we performed a detailed behavioral characterization of *Scn2a*^+/−^ mice comparing the juvenile/adolescent period of development and adulthood. We used tasks relevant to ASD and the different comorbidities frequently found in this disorder, such as anxiety or intellectual disability. Our data demonstrate that young *Scn2a*^+/−^ mice display autistic-like phenotype associated to impaired memory and reduced reactivity to stressful stimuli. Interestingly, these dysfunctions are attenuated with age since adult mice show only communicative deficits. Considering the clinical data available on patients with loss-of-function *SCN2A* mutations, our results indicate that *Scn2a*^+/−^ mice constitute an ASD model with construct and face validity during the juvenile/adolescent period of development. However, more information about the clinical features of adult carriers of *SCN2A* mutations is needed to evaluate comparatively the phenotype of adult *Scn2a*^+/−^ mice.

## Introduction

Mutations of the *SCN2A* gene, which encodes the voltage-gated sodium channel Na_v_1.2, have been associated to a wide spectrum of epileptic disorders ranging from mild forms, as benign familial neonatal-infantile seizures (BFNIS), to very severe ones, as early-infantile epileptic encephalopathies, Ohtahara syndrome or West syndrome^1–6^. While BFNIS is caused mostly by inherited missense mutations, epileptic encephalopathies have been associated with *de novo* truncating or missense mutations. More recently, *de novo SCN2A* mutations have also been identified in patients with intellectual disability (ID), autism spectrum disorder (ASD) or schizophrenia without overt epilepsy, enlarging thus the spectrum of *SCN2A*-associated disorders^7–10^. *In vitro* electrophysiological studies have shown that the different epileptic phenotypes may be caused by either gain- or loss-of-function *SCN2A* mutations, whereas it has been proposed that ASD, and possibly also ID or schizophrenia, arise from loss-of-function mutations^11–16^.

Na_v_1.2 channels are widely expressed in many structures of the mammalian brain, including neocortex, hippocampus, striatum, globus pallidus and cerebellum^17,18^. They are important for neuronal excitability and synaptic depolarization leading to neurotransmitter release. In fact, Na_v_1.2 channels are present both in excitatory and inhibitory neurons and largely associated with axons (axon initial segment and nodes of Ranvier) and presynaptic terminals^17–20^, although they have been observed also in somatodendritic compartments of pyramidal glutamatergic neurons^17,21^. Notably, expression of Na_v_1.2 channels in axon initial segments of glutamatergic neurons shows a developmental pattern: Na_v_1.2 is the only subtype present in the first post-natal days, and is partially replaced by Na_v_1.6 during maturation, at around the third week of age in mice^13,22^. These spatial and temporal patterns of expression add to the complexity of genotype-phenotype relationships associated with *SCN2A* mutations.

Recent studies have shown that adult heterozygous *Scn2a* knock-out mice (*Scn2a*^+/−^) display a relatively mild epileptic phenotype of absence-like seizures^23,24^, as well as delayed spatial learning associated with altered hippocampal replay^25^. More recently, additional behavioral alterations have been reported in these mice, in particular hyperactivity and impaired fear extinction^26^. Because *SCN2A* haploinsufficiency has been proposed to be linked to ASD, in the present study we undertook a detailed behavioral characterization of *Scn2a*^+/−^ mice focused on behavioral tasks relevant to ASD and the different comorbidities frequently found in this disorder, such as anxiety or intellectual disability. Importantly, since ASD appears during early childhood, we have analyzed the behavioral phenotype of these mice at two developmental stages, the juvenile/adolescent period and the adulthood.

## Results

### Both young and adult Scn2a^+/−^ mice display reduced ultrasonic vocalizations but mild or no impairment of social interaction behavior

**Figure 1:**
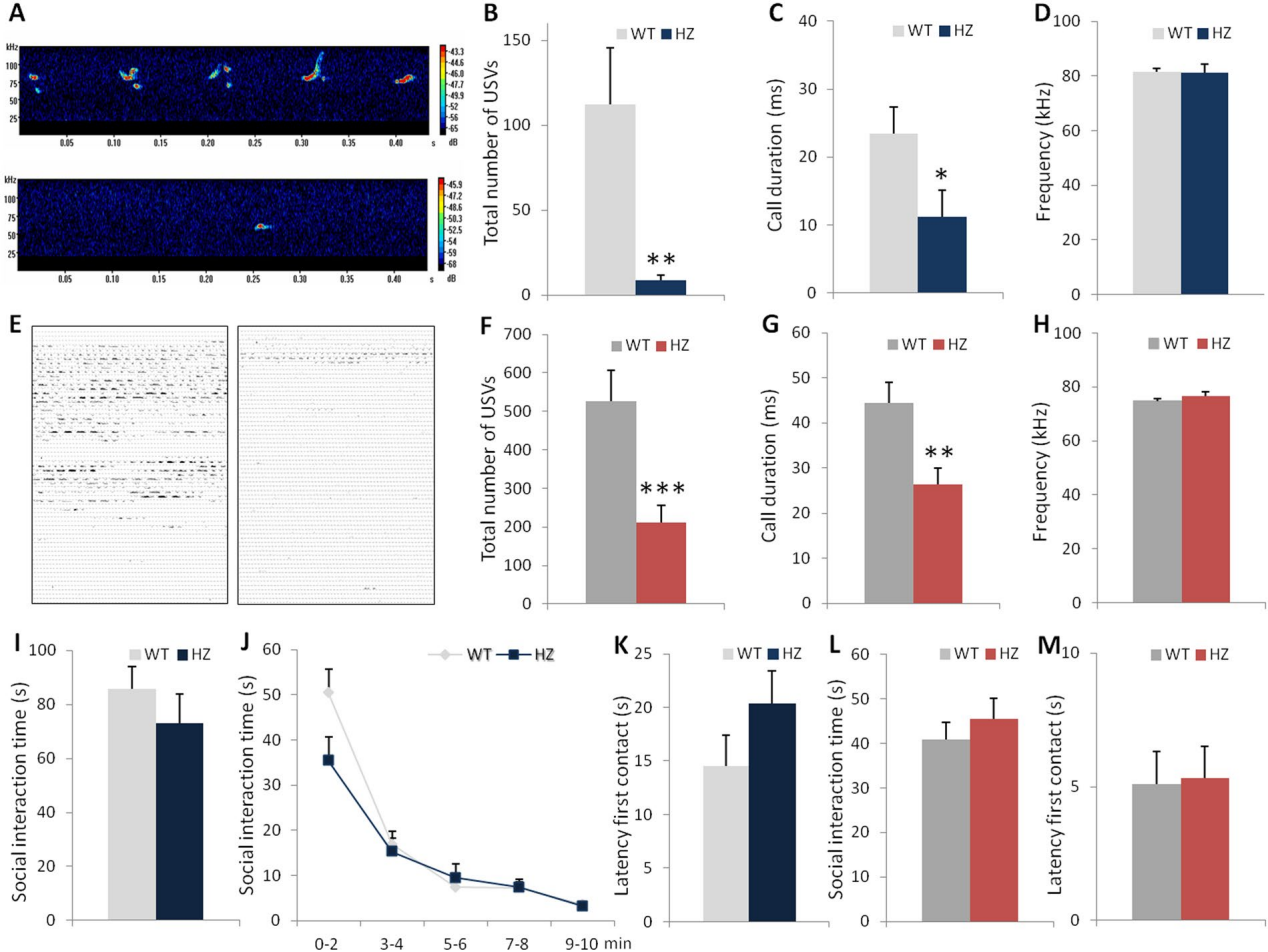
Young and adult *Scn2a*^+/−^ mice display reduced social communication but mild or no impairment of social interactions. (**A**–**D**) Social communication in young mice (wild-type (WT) n = 9 pairs; *Scn2a*^+/−^ n = 7 pairs). (**A**) Representative sound spectrograms of ultrasonic vocalizations (USV) (pseudocolors indicate intensity of calls in dB) emitted by pairs of unfamiliar WT mice (higher panel) or *Scn2a*^+/−^ mice (lower panel). Total number (**B**), mean duration (**C**) and average frequency of USV (**D**) in pairs of unfamiliar male mice of the same genotype (Welch’s t-test or Mann-Whitney test: *p ≤ 0.05, **p < 0.01 vs WT). (E-H) Social communication in adult mice. (**E**) Representative USV recordings emitted by WT (left panel) or *Scn2a*^+/−^ (right panel) male mice in the presence of an estrus female during the 3-min session. Total number (**F**), mean duration (**G**) and average frequency of USV (**H**) emitted by adult mice (Welch’s or Student’s t- test: **p < 0.01, ***p < 0.005 vs WT). (**I**) Total time spent in social interaction initiated by the test mouse (WT or *Scn2a*^+/−^) toward an unfamiliar WT stimulus mouse during the 10-min session in young animals. (**J**) Time-course of social interactions. (**K**) Latency for first social contact in young mice. (**L**) Total social interaction time initiated by the test mouse (WT or *Scn2a*^+/−^) in adult animals during the 5-min session. (**M**) Latency for first social contact in adults. (**E**–**M**: WT n = 17; *Scn2a*^+/−^ n = 14).

One diagnostic criterion for ASD is the presence of deficits in social communication and social interactions^27^. Since mice communicate by emitting USV during social play behavior in the juvenile period and during courtship episodes between males and females in adulthood, we recorded USV in these different social situations in young and adult mice. Figure 1A–C show that pairs of unfamiliar young *Scn2a*^+/−^ male mice display a significant decrease in the total number of ultrasonic calls produced (unpaired Welch’s t-test, t(8) = 3.44, p = 0.0083; Fig. 1A,B) and in the mean duration of each call (Mann-Whitney test, U = 62, p = 0.0504; Fig. 1C) during the 5-min session compared to pairs of wild-type mice, indicating a deficit in social communication in heterozygous mice. The average frequency of ultrasonic calls did not differ between genotypes (unpaired Student’s t-test, t(14) = 0.69, p = 0.9172; Fig. 1D). Similarly to young mice, adult *Scn2a*^+/−^ males emitted significantly less USV in the presence of an estrous female compared to *Scn2a*^+/+^ mice during the 3-min session (unpaired Welch’s t-test, t(26) = 3.58, p = 0.0014; Fig. 1E,F). The mean duration of calls emitted by adult heterozygous males was significantly shorter than those produced by control littermates (unpaired Student’s t-test, t(27) = 2.91, p = 0.0072; Fig. 1G), whereas the average frequency of calls was similar between genotypes (unpaired Student’s t-test, t(27) = −1.2, p = 0.2405; Fig. 1H).

Then, we investigated social behavior in freely moving pairs of unfamiliar male mice. In the reciprocal social interaction test with young mice, no significant differences were found between genotypes in the total time spent in social interaction initiated by the test mouse toward an unfamiliar wild-type stimulus mouse during the 10-min session (unpaired Student’s t-test, t(29) = 0.54, p = 0.5952; Fig. 1I). We further analyzed social interactions by bins of 2 min. Both genotypes exhibited a gradual reduction in the amount of time engaged in social interaction over time (two-way repeated measures ANOVA, F(1, 29) = 0.736, p = 0.3981 for genotype; F(4, 116) = 80.7, p < 0.0001 for time; F(4, 116) = 3.03, p = 0.0202 for genotype x time interaction; Fig. 1J). However, the significant genotype by time interaction indicates that the time course of social interactions is genotype specific. Indeed, as can be seen in Fig. 1J, young male *Scn2a*^+/−^ mice spent less time in social interaction compared to wild-type littermates during the first two minutes of the session. Heterozygous mice, however, did not differ significantly from wild-type mice in the latency to initiate the first social contact (unpaired Student’s t-test, t(29) = −1.44, p = 0.1601; Fig. 1K). In contrast, adult male *Scn2a*^+/+^ and *Scn2a*^+/−^ mice did not show any significant differences in the amount of time engaged in social interactions initiated by the test mouse toward the wild-type stimulus mouse during the 5-min session (unpaired Student’s t-test, t(29) = −0.80, p = 0.4304; Fig. 1L) or the latency to establish the first social contact (unpaired Student’s t-test, t(29) = −0.12, p = 0.9051; Fig. 1M). The time spent in social interaction during the first 2 min of the test was 32.19 ± 3.04 s for *Scn2a*^+/+^ and 38.45 ± 3.85 s for *Scn2a*^+/−^ adult mice.

**Figure 2:**
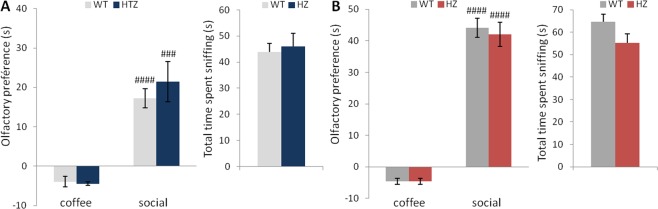
Young and adult *Scn2a*^+/−^ mice show intact olfactory discrimination and preference for social odors. (**A**) Time spent sniffing social or non-social odors (coffee) (with time sniffing water substracted) and total time investigating the three odors (water, coffee, social) in young mice. (**B**) Olfactory preference and total time spent sniffing the three odors in adult mice. (Paired t-test: ^###^p < 0.0005, ^####^p < 0.0001 vs coffee odor for WT and *Scn2a*^+/−^mice; n = 14/genotype).

Collectively, these findings demonstrate that *Scn2a*^+/−^ mice show persistent difficulties in social communication until adulthood. These deficits are unlikely due to loss of olfaction, since our data (Fig. 2) show that young and adult *Scn2a*^+/−^ mice are able to discriminate between social and non-social odors. Indeed, young mice from both genotypes displayed more interest in social odors compared to non-social odors (two-way repeated measures ANOVA, F(1, 26) = 0.37, p = 0.5466 for genotype; F(1, 26) = 70.76, p < 0.0001for odor; F(1, 26) = 0.71, p = 0.4055 for genotype × odor interaction; paired t-test, social vs coffee: t(13) = −7,98, p < 0.0001 for *Scn2a*^+/+^ and t(13) = −5,26, p = 0.0002 for *Scn2a*^+/−^) and did not show any significant differences in the total time spent investigating the three odors (unpaired Student’s t-test, t(26) = −0.36, p = 0.7118; Fig. 2A). Similarly, in adulthood, *Scn2a*^+/+^ and *Scn2a*^+/−^ mice showed comparable performance in the odor discrimination task (two-way repeated measures ANOVA, F(1, 26) = 1.91, p = 0.1792 for genotype; F(1, 26) = 528.2, p < 0.0001 for odor; F(1, 26) = 1.30, p = 0.2646 for genotype × odor interaction; paired t-test, social vs coffee: t(13) = −19.4, p < 0.0001 for *Scn2a*^+/+^ and t(13) = −13.94, p < 0.0001 for *Scn2a*^+/−^) without significant change in the total time spent investigating the different odors (unpaired Student’s t-test, t(26) = 1.80, p = 0.0827; Fig. 2B). This is consistent with the results of a recent study^28^, which showed that deletion of Na_v_1.2 channels in granule cells of the olfactory bulb did not prevent the mice from discriminating odors, but slowed the time needed to discriminate between highly similar odors.

### Stereotyped and repetitive behaviors are present in young but not adult Scn2a^+/−^ mice

**Figure 3:**
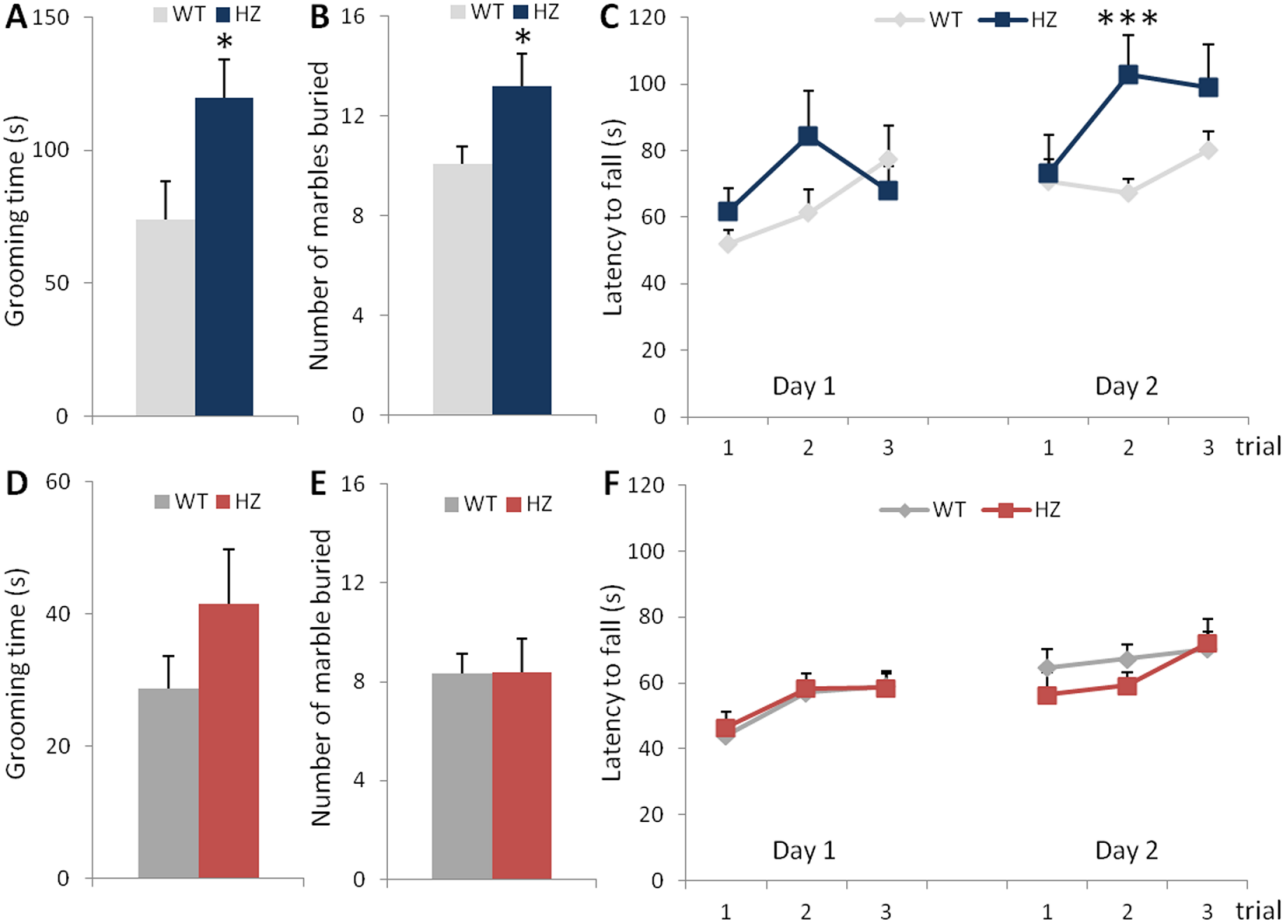
Motor stereotypies are present in young but not adult *Scn2a*^+/−^ mice. (**A**–**C**) Stereotyped behaviors in young mice. (**A**) Time spent in self-grooming (Student’s t-test: *p < 0.05), (**B**) number of marbles buried (Student’s t-test: *p < 0.05 vs WT) (**C**) and latency before falling in the rotarod test (Bonferroni’s *post-hoc* test: ***p < 0.005 vs WT). (**D**–**F**) Stereotyped behaviors in adult mice. (**D**) Time spent in self-grooming, (**E**) number of marbles buried and (**F**) latency before falling in the rotarod test. (WT, n = 17; *Scn2a*^+/−^, n = 14).

A further criterion for the diagnosis of ASD is the presence of restricted, repetitive and stereotyped patterns of behavior^27^. We therefore investigated if young male *Scn2a*^+/−^ mice display stereotyped motor movements such as self-grooming behavior. Figure 3A shows that young *Scn2a*^+/−^ mice spent significantly more time in self-grooming compared to young *Scn2a*^+/+^ mice during a 10-min session in an open-field arena (unpaired Student’s t-test, t(29) = −2.24, p = 0.0328). We also used the marble burying test (Fig. 3B), in which young *Scn2a*^+/−^ mice showed a significant increase in the number of buried marbles compared to wild-type littermates (unpaired Student’s t-test, t(29) = −2.26, p = 0.0313), further supporting the presence of repetitive and perseverative behavior in these mutant mice^29^.

Then, we analyzed motor behavior in the rotarod test, since repetitive behaviors are not only innate but can also be acquired during training. Indeed, enhanced acquisition of repetitive motor routines in the rotarod test have been described in several ASD mouse models such as *Pten*^−/−^, *Cntnap2*^−/−^, *Nl3*^−/−^ or *Chd8*^+/−^ mice^30–33^. Young *Scn2a*^+/−^ mice spent more time than wild-type mice on the rotarod before falling (Fig. 3C). From the second day of the test, performance of heterozygous mice improved significantly, indicating an increased acquired motor learning (two-way repeated measures ANOVA, Day 1: F(1, 29) = 0.78, p = 0.3846 for genotype; F(2, 58) = 3.26, p = 0.0456 for trial; F(2, 58) = 2.53, p = 0.0885 for genotype × trial interaction; Day 2: F(1, 29) = 4.31, p = 0.0470 for genotype; F(2, 58) = 3.45, p = 0.0383 for trial; F(2, 58) = 2.94, p = 0.0609 for genotype × trial interaction; Bonferroni’s *post-hoc* test: trial 2: p = 0.0033).

In adulthood, male *Scn2a*^+/−^ mice, unlike young mice, did not show a significant increase in self-grooming time compared to *Scn2a*^+/+^ mice (Mann-Whitney test, U = 86, p = 0.3152; Fig. 3D). Moreover, wild-type and mutant adult mice did not differ in the number of buried marbles (unpaired Student’s t-test, t(29) = −0.003, p = 0.9978; Fig. 3E). Additionally, adult mice from both genotypes exhibited comparable performances during the two days of the rotarod test (two-way repeated measures ANOVA, Day 1: F(1, 29) = 0.09, p = 0.7714 for genotype; F(2, 58) = 7.09, p = 0.018 for trial; F(2, 58) = 0.07, p = 0.9312 for the interaction; Day 2: F(1, 29) = 0.66, p = 0.4232 for genotype; F(2, 58) = 3.13, p = 0.0505 for trial; F(2, 58) = 0.87, p = 0.4235 for the interaction; Fig. 3F), suggesting normal coordination and motor learning.

Altogether, these results demonstrate that repetitive stereotyped behaviors are key features of young but not adult *Scn2a*^+/−^ mice.

### Hyporeactivity to negative emotional stimuli is observed in young but not adult Scn2a^+/−^ mice

**Figure 4:**
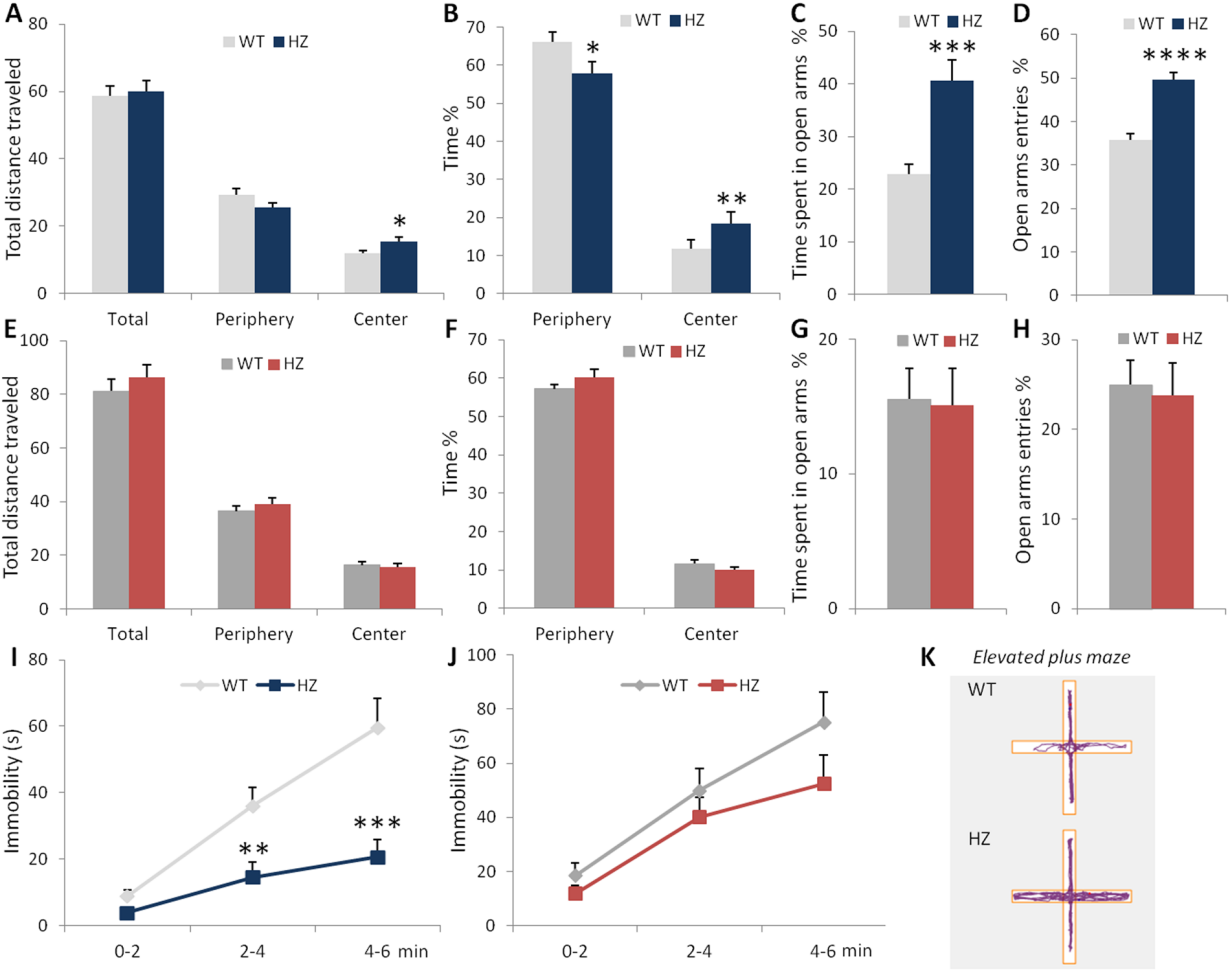
Young, but not adult, *Scn2a*^+/−^ mice are less anxious and less resigned. (**A**–**D**) Anxiety-related behaviors in young mice evaluated in the open-field test (**A,B**) and the elevated plus-maze test (**C**,**D**). (**A**) Distance traveled (m) per zone during the 30-min open-field test session (Student’s t-test: *p < 0.05). (**B**) Percentage of time spent in the periphery and the central area of the open-field (Mann-Whitney test: *p < 0.05, **p < 0.01 vs WT). Percentage of time spent (**C**) and of number of entries (**D**) in the open arms of the elevated plus-maze (Welch’s t-test ou Mann-Whitney test: ***p < 0.001, ****p < 0.0001 vs WT). (**E**–**H**) Anxiety-related behaviors in adult mice in the open-field (**A**,**B**) and the elevated plus-maze tests (**C**,**D**). (**A**–**H**: WT, n = 17; *Scn2a*^+/−^, n = 14). (**I**–**J**) Depression-related behavior assessed in the tail suspension test by the time spent in immobility during the 6-min session for young (Bonferroni’s *post-hoc* test: **p < 0.01, ***p < 0.005; WT, n = 16; *Scn2a*^+/−^, n = 14) (**I**) and adult (WT, n = 16; *Scn2a*^+/−^, n = 12) (**J**) mice. Note that 1 young and 1 adult wild-type mice and 2 adult mutant mice climbed their tail and were removed for results. (**K**) Representative track plots of young mice during the elevated plus-maze test (open arms are in the horizontal axis).

Because anxiety and depression are among the most frequently diagnosed comorbidities in patients with ASD^34^, we assessed anxiety- and depression-related behavior in *Scn2a*^+/−^ mice. In the open-field test (Fig. 4A), the total distance traveled by young male *Scn2a*^+/−^ mice was not significantly different from that of control *Scn2a*^+/+^ littermates (Mann-Whitney test, U = 102, p = 0.5125), reflecting a normal exploratory behavior in heterozygous mice. However, young *Scn2a*^+/−^ mice showed a significant increase in the distance traveled (unpaired Student’s t-test, t(29) = −2.43, p = 0.0214) and time spent (Mann-Whitney test, U = 54, p = 0.0105) in the central area, along with a decrease in the time spent in the periphery (Mann-Whitney test, U = 180, p = 0.0163), compared to wild-type mice (Fig. 4B). In the elevated plus maze test (Fig. 4C,D,K), young *Scn2a*^+/−^ mice displayed a significant higher percentage of time spent (Welch’s t test, t(19) = −4.13, p = 0.0005) and number of entries (Mann-Whitney test, U = 6, p < 0.0001) in the open arms compared to wild-type littermates. As in the open-field test, no difference in locomotor activity was observed between the two genotypes as shown by the total distance traveled as well as the total number of entries in closed/open arms (Table 1). In the tail suspension test (Fig. 4I), young *Scn2a*^+/−^ mice spent significantly less time immobile than WT mice during the 6-min session (two-way repeated measures ANOVA, F(1, 28) = 13.55, p = 0.0010 for genotype; F(2, 56) = 12.26, p < 0.0001 for time; F(2, 56) = 3.23, p = 0.0305 for genotype × time interaction; Bonferroni’s *post hoc* test, 2–4 min: p = 0.0051; 4–6 min: p = 0.0023), suggesting that young heterozygous mice are less resigned. Taken together, these results indicate that young *Scn2a*^+/−^ mice display reduced emotional reactivity to negative stimuli.

**Table 1:** Additional parameters in the elevated plus maze test.

	Young mice		p-value	Adult mice		p-value
	*WT*	*HZ*		*WT*	*HZ*	
Total distance traveled (m)	6.51 ± 0.3	6.26 ± 0.4	0.5917	8.53 ± 0.4	8.25 ± 0.6	0.7022
Number of total entries in open and closed arms	18.7 ± 0.9	19.2 ± 1.3	0.5493	21.6 ± 1.3	21.7 ± 1.9	0.9553
Distance moved in open arms (m)	1.59 ± 0.1	2.38 ± 0.2	0.0202	1.29 ± 0.2	1.21 ± 0.3	0.5920
Distance moved in closed arms (m)	3.71 ± 0.1	2.69 ± 0.2	<0.0001	4.80 ± 0.3	4.68 ± 0.3	0.7719
Velocity in open arms (cm/s)	2.91 ± 0.1	2.61 ± 0.1	0.1586	3.73 ± 0.2	3.79 ± 0.4	0.8176
Velocity in closed arms (cm/s)	2.04 ± 0.1	2.05 ± 0.2	0.9472	2.58 ± 0.1	2.73 ± 0.2	0.5661

Comparisons between genotypes were performed using unpaired Student’s t-test or Mann-Whitney test.

In adulthood, male *Scn2a*^+/+^ and *Scn2a*^+/−^ mice showed similar exploratory/locomotor behavior in the open-field (Mann-Whitney test, U = 78, p = 0.1079; Fig. 4E) and elevated-plus maze tests (Table 1), as observed during the juvenile/adolescent period of development. However, in contrast to young *Scn2a*^+/−^ mice, no significant genotype effect was detected on the percentage of time spent in the center area of the open-field (unpaired Student’s t-test, t(29) = 1.36, p = 0.1846) or in the open arms of the plus maze (unpaired Student’s t-test, t(29) = 0.13, p = 0.9001), indicating that adult heterozygous mice do not display lower anxiety (Fig. 4F–H; Table 1). Similarly, in the tail suspension test (Fig. 4J), *Scn2a*^+/+^ and *Scn2a*^+/−^ adult mice did not show any significant differences in the time spent immobile, although a tendency to decreased immobility time was observed for *Scn2a*^+/−^ mice at the end of the 6-min session (two-way repeated measures ANOVA main, F(1, 26) = 3.15, p = 0.0875 for genotype (borderline significance); F(2, 52) = 5.06, p = 0.0098 for time; F(2, 52) = 0.72, p = 0.4892 for the interaction).

Taken together, these data indicate that the reduced sensitivity to stressful stimuli found in young *Scn2a*^+/−^ mice does not persist in adulthood. Importantly, it is unlikely that the hyporeactivity to negative emotional stimuli can contribute to the reduced social communication and stereotyped behaviors that we observed in young mutant mice since, on the contrary, increased anxiety results in lower social interactions and excessive motor stereotypies^29,35,36^.

### Young Scn2a^+/−^ mice display impaired memory functions whereas adult Scn2a^+/−^ mice show a tendency for slower spatial learning abilities

**Figure 5:**
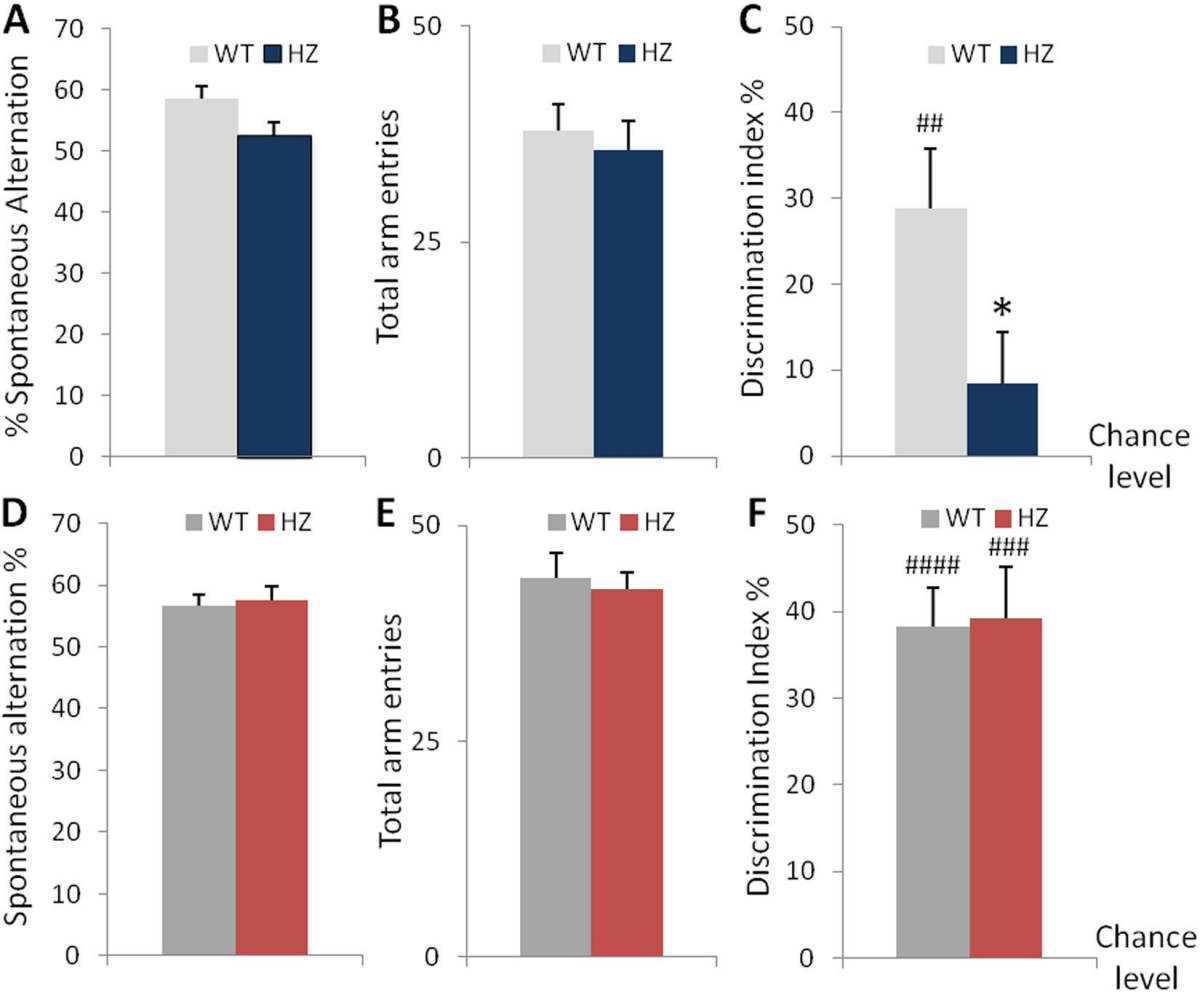
Memory performance is impaired in young but not adult *Scn2a*^+/−^ mice. (**A**,**B**) Spatial working memory and (**C**) recognition memory in young mice. (**A**) Percentage of spontaneous alternations (Student’s t-test: p = 0.059) and (**B**) total number of visits in the Y maze test (WT, n = 17; *Scn2a*^+/−^, n = 14). (**C**) Discrimination index in the novel object recognition memory task (Student’s t-test: *p < 0.05 vs WT; one-sample t-test: ^##^p < 0.001vs chance level; WT, n = 15; *Scn2a*^+/−^, n = 12). (**D**,**E**) Percentage of spontaneous alternations and total number of visits in the Y maze test in adult mice (WT, n = 17; *Scn2a*^+/−^, n = 14). (**F**) Discrimination index in the novel object recognition memory task in adult mice (one-sample t-test: ^###^p < 0.0005, ^####^p < 0.0001vs chance level; WT, n = 15; *Scn2a*^+/−^, n = 12). (**C**,**F**) Note that 2 WT and 2 mutant mice at both ages did not reach the criterion of minimal level of object exploration during the second session (see Methods) and were removed from the results.

About 45% of subjects with autistic disorders have some degree of intellectual disabilities^34,37^. We therefore evaluated the presence of cognitive deficits in young and adult *Scn2a*^+/−^ mice using different memory tests. We first assessed spontaneous alternation behavior in a Y maze. Young *Scn2a*^+/−^ mice displayed a decrease in the percentage of spontaneous alternations at borderline significance compared to wild-type mice (unpaired Student’s t-test, t(29) = 1.96, p = 0.0593; Fig. 5A), without change in the total number of visits (unpaired Student’s t-test, t(29) = 0.83, p = 0.4134; Fig. 5B), suggesting that there are no major or very mild impairments in spatial working memory in heterozygous mice. In the novel object recognition task (Fig. 5C), young *Scn2a*^+/+^ mice spent more time exploring the novel object over the familiar object during the 5-min retention test, as indicated by a discrimination index higher than chance level (one sample t-test, t(14) = 4.08, p = 0.0011). In contrast, the discrimination index was not above chance level for young *Scn2a*^+/−^ mice (one sample t-test, t(11) = 1.39, p = 0.1907), which failed to display a preference for the novel object compared to wild-type littermates (unpaired Student’s t-test, t(25) = 2.13, p = 0.0435). The total time spent exploring the novel and the familiar objects did not differ between genotypes (Table 2), indicating that the reduced discrimination index observed in young *Scn2a*^+/−^ mice was not related to a decrease in exploration, in accordance with the normal exploratory behavior that we observed in the open-field test.

**Table 2:** Additional measures of exploration in the novel object recognition task.

	Young mice		p-value	Adult mice		p-value
	*WT*	*HZ*		*WT*	*HZ*	
Time necessary to explore for 20 s the two identical objects during the acquisition phase (first trial) (s)	210.4 ± 23.1	218.8 ± 20.2	0.7914	242.0 ± 20.7	238.6 ± 23.1	0.9144
Total time spent exploring both objects (novel and familiar) during the retention test (second trial) (s)	27.15 ± 2.6	24.22 ± 2.1	0.4109	38.11 ± 1.3	35.31 ± 3.6	0.5549

Comparisons between genotypes were performed using unpaired Student’s t-test.

In adulthood, *Scn2a*^+/−^ mice did not show any significant differences compared to *Scn2a*^+/+^ mice in the percentage of spontaneous alternations (unpaired Student’s t-test, t(29) = −0.32, p = 0.7528; Fig. 5D) or the total number of visits in the Y maze test (unpaired Welch’s t-test, t(26) = 0.33, p = 0.7433; Fig. 5E). Furthermore, in the object recognition task (Fig. 5F), both wild-type and heterozygous adult mice exhibited a clear preference for the novel object during the retention test, as assessed by a discrimination index significantly above chance level (one sample t-test, t(14) = 6.94, p < 0.0001 for *Scn2a*^+/+^; t(11) = 5.08, p = 0.0004 for *Scn2a*^*+/*^). Both genotypes displayed similar discrimination index (unpaired Student’s t-test, t(25) = −0.10, p = 0.9202) and amount of time spent exploring the two objects (Table 2).

**Figure 6:**
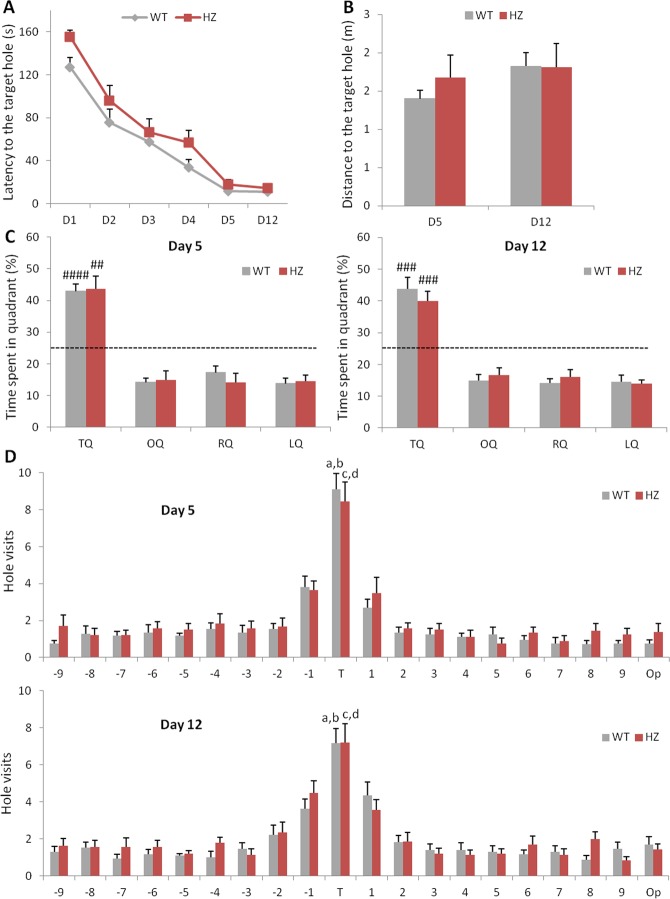
Adult WT and *Scn2a*^+/−^ mice show comparable spatial memory performance in the Barnes maze test. (**A**) During the training days (day1–4), *Scn2a*^+/−^ mice showed a trend towards a significant slower acquisition latencies, but, during the probe trials on days 5 and 12, the latency and (**B**) the distance traveled before finding the target hole did not differ between genotypes. (**C**) Percentage of time spent in the correct quadrant, where the target hole was located, and in the other quadrants (one sample t-test, ^##^p < 0.005, ^###^p < 0.0005, ^####^p < 0.00001 vs chance level). (**D**) Number of visits to each hole on day 5 (Paired t-test: ^a^p < 0.0005 vs -1 hole, ^b^p < 0.0001 vs +1 hole for WT; ^c^p < 0.005 vs -1 hole, ^d^p < 0.0005 vs +1 hole for *Scn2a*^+/−^) and day 12 (Paired t-test: ^a^p = 0.001 vs -1 hole, ^b^p = 0.005 vs +1 hole for WT; ^c^p < 0.05 vs -1 hole, ^d^p < 0.01 vs +1 hole for *Scn2a*^+/−^). Dashed line indicates chance performance (25%). T = Target, O = Opposite, L = Left, R = Right. (WT, n = 17; Scn2a^+/−^, n = 14).

In order to better disclose learning and memory dysfunctions of adult *Scn2a*^+/−^ mice, we used the Barnes maze test. The latency to reach the escape box decreased over the training days (Day1–4) for both genotypes (Fig. 6A). Although adult *Scn2a*^+/−^ mice showed a trend towards significant slower acquisition latencies on day1–4 (two-way repeated measures ANOVA, F(1, 29) = 3.56, p = 0.0693 for genotype (borderline significance); F(5, 145) = 90.04, p < 0.0001 for day; F(5, 145) = 1.1, p = 0.3813 for genotype × day interaction), their performances were comparable to wild-type mice during the probe tests on days 5 and 12. Indeed, Fig. 6B shows that the distance traveled before finding the target hole did not differ between genotypes on day 5 (unpaired Welch’s t-test, t(22) = −0.53, p = 0.6024) or day 12 (unpaired Student’s t-test, t(29) = −0.39, p = 0.7011). Moreover, no significant genotype differences were found in the percentage of time spent in the correct quadrant, where the target hole was located, during the probe trials (two-way repeated measures ANOVA, Day 5: F(1, 29) = 2.53, p = 0.1226 for genotype; F(3, 87) = 44.18, p < 0.0001 for quadrant; F(3, 87) = 0.19, p = 0.9030 for genotype × quadrant interaction; Day 12: F(1, 29) = 1.02, p = 0.3215 for genotype; F(3, 87) = 46.72, p < 0.0001 for quadrant; F(3, 87) = 0.47, p = 0.7007 for the interaction; Fig. 6C). For each genotype, the percentage of time spent in the target quadrant was significantly higher than chance level on day 5 (one sample t-test, t(16) = 8.18, p < 0.00001 for *Scn2a*^+/+^; t(13) = 4,27, p = 0.0009 for *Scn2a*^+/−^) and day 12 (one sample t-test, t(16) = 5.15, p < 0.0001 for *Scn2a*^+/+^; t(13) = 4.72, p < 0.0005 for *Scn2a*^+/−^). Finally, both genotypes visited the target hole more often compared to the adjacent holes on day 5 (paired t-test, target vs −1 hole: t(16) = 4.55, p = 0.0003; target vs +1 hole: t(16) = 6.56, p < 0.0001 for *Scn2a*^+/+^ and target vs -1 hole: t(13) = 4.08, p = 0.0013; target vs +1 hole: t(13) = 5.06, p = 0.0002 for *Scn2a*^+/−^) and day 12 (paired t-test, target vs −1 hole: t(16) = 4.03, p = 0.0010; target vs +1 hole: t(16) = 3.24, p = 0.0051 for *Scn2a*^+/+^ and target vs −1 hole: t(13) = 2.62, p = 0.0211; target vs +1 hole: t(13) = 3.09, p = 0.0086 for *Scn2a*^+/−^; Fig. 6D).

Overall, these data suggest that young *Scn2a*^+/−^ mice exhibit impairment in recognition memory and a tendency to reduced spatial working memory, while adult heterozygous mice have intact spatial working and long-term memory, as well as intact object recognition memory, showing only a tendency to slower spatial learning.

## Discussion

*SCN2A* mutations have been identified as one of the most significant risk factors for ASD^8,38^. It has been proposed that these mutations, which are often missense, cause nearly complete loss-of-function of Na_v_1.2^15^. Our study demonstrates that Na_v_1.2 haploinsufficiency in mice -modeling heterozygous Na_v_1.2 complete loss-of-function associated with mutations identified in humans- causes a self-limited autistic-like phenotype, which is attenuated with age. Thus, young *Scn2a*^+/−^ mice exhibit deficits in social communication and interaction as well as repetitive stereotyped behaviors, recapitulating core symptoms of ASD, whereas adult heterozygous mice display only reduced social communication. In addition to autistic-like behaviors, young mutant mice display impaired memory and low reactivity to stressful stimuli, alterations that in our study do not persist in adulthood.

The genetic studies that identified loss-of-function *SCN2A* mutations associated with ASD without overt epilepsy were performed on patients aged from 3 to 17 years^7–9,16,39–41^. Among these studies, a few reported other symptoms than the core ASD features. Intellectual disability, ranging from moderate to severe, was the most frequently observed comorbidity in these children or adolescents with ASD^7,9,16,40,41^. Abnormal gait and/or fine coordination was observed in two autistic children^9,40^, attention deficit hyperactivity disorder in one adolescent^41^, and obsessive-compulsive disorder associated to anxiety in one child with Asperger’s syndrome^40^. The autistic-like behaviors and comorbid cognitive deficits that we observed in *Scn2a*^+/−^ mice during the juvenile/adolescent period of development are therefore consistent with the symptoms described in these young patients. However, no clinical information, to our knowledge, is available about adult carriers of *SCN2A* mutations suffering of non-syndromic ASD. We could not, therefore, evaluate whether adult *Scn2a*^+/−^ mice exhibit behavioral phenotype similar to those observed in humans, especially the attenuation of autistic symptoms. Of particular interest, autistic symptoms follow heterogeneous developmental trajectories (six patterns were identified by Fountain *et al*.^42^) and good outcome has been described in adulthood for a subset of individuals diagnosed with autism in childhood^42–45^. Such longitudinal studies in patients with loss-of-function *SCN2A* mutations may be useful to correlate the mild phenotype that we observed in adult heterozygous mice.

Young *Scn2a*^+/−^ mice display strong deficit in social communication but mild impairment in social interaction associated to several motor stereotypies. It is not excluded that, in other experimental conditions (long time isolation before test, pairs of mutant mice) or in other tests like the three-chamber sociability test, social interaction deficits might be more pronounced. Importantly, if lack of social interactions is a symptom shared by other disorders such as schizophrenia or depression, reduced social communication associated to repetitive behaviors early in childhood are specific to ASD. Dysfunction in striatal circuits, especially cortico-striatal pathways, has been postulated to underlie autistic behaviors^46,47^. A marked reduction in Na_v_1.2 expression in these circuits during development might therefore contribute to the autistic phenotype seen in young *Scn2a*^+/−^ mice. Interestingly, it has been recently shown that cortico-striatal pathways are involved in the generation of absence-like seizures in *Scn2a*^+/−^ mice^24^.

Intriguingly, young mutant mice show reduced anxiety in the open-field and plus maze tests along with decreased resignation in the tail suspension test. Autistic patients often exhibit anxiety or depression and some ASD mouse models, such as *Shank3B*^−/−^, *Chd8*^+/−^ or *En2*^−/−^ mice, display anxiety-like^33,48^ or depression-like^49^ phenotypes. Interestingly, our results are very similar to those described in BTBR mice^50,51^. This inbred strain with autistic-like behaviors displays lower levels of anxiety in the elevated plus maze or zero maze tests (although these results have not been always replicated), and low depression-related behavior in the tail suspension test. These behaviors were associated to alterations in the hypothalamic-pituitary-adrenal (HPA) axis. It is therefore tempting to hypothesize that young *Scn2a*^+/−^ mice have similar altered HPA axis activity which could explain their lower sensitivity to stressful stimuli. Altered HPA axis activation might be the consequence of decreased Na_v_1.2 expression in hypothalamic or limbic circuits involved in the regulation of this axis, such as activatory pathways from the amygdala, where Na_v_1.2 channels are expressed^23^. Thus, decreased activity of afferents from the amygdala would reduce the HPA axis response to stressful environments. Of particular interest, emotional reactivity to negative stimuli early in life is predominantly influenced by the amygdala and is regulated at later stages of development by the prefrontal cortex, which can exert top-down control on the amygdala, leading to improvements in emotional regulation and anxiety^52,53^.

In addition to lower reactivity to stressful stimuli, young *Scn2a*^+/−^ mice display cognitive deficits as evidenced by altered mnesic functions. The mildly reduced spatial working memory (borderline significance) and the impaired object recognition memory suggest dysfunctions in hippocampal and prefrontal or perirhinal cortex circuits, respectively^54,55^. These memory tests are based on the spontaneous tendency of rodents to explore novelty. The deficits observed are unlikely due to neophobia since young mutant mice did not show any decrease in overall exploration of the maze or objects in these tasks. On the contrary, lack of interest for novelty, which may resemble to restricted interests described in ASD patients, might contribute to memory deficits. Alternatively, alteration in attentional processes in young *Scn2a*^+/−^ mice may interfere with memory performance. Indeed, attention-deficit hyperactivity disorder (ADHD) is a frequent comorbid trait in ASD patients^34,37^ and has been reported in one autistic adolescent carrier of loss-of-function *SCN2A* mutation^41^.

Unexpectedly, in adulthood, much milder behavioral abnormalities were observed in *Scn2a*^+/−^ mice, since only social communication was significantly reduced. Mutant mice also showed a tendency for slower spatial learning in the Barnes maze, consistent with the delayed spatial learning reported recently by Middleton *et al*.^25^ in the same test. Importantly, adult heterozygous mice do not display impairments in memory performance in this task and the other memory tasks used (i.e., spontaneous alternation and object recognition). A tendency to decreased immobility time in the tail suspension test was also detected in adult mutant mice in agreement with the very recent study of Tatsukawa *et al*.^26^. The absence of social behavior deficits that we observed in the direct social interaction task is further consistent with the results reported in that study. However, Tatsukawa *et al*.^26^ showed that adult heterozygous mice exhibited a mild hyperactivity in the open-field test, a lower level of anxiety in the plus maze test (but the contrary in the light-dark box test) and a reduced performance in the rotarod task, abnormalities that we did not detect using the same tests. The differences between our results and those of Tatsukawa *et al*.^26^ may be explained by different housing conditions (single versus group-housed mice with mixed genotypes in the present study), experimental conditions (luminosity, duration of tests) and protocols used (one day with 6 trials versus 2 days tests preceded by a training session for the rotarod test in our study)^56^. Another known source of variability in the outcome of behavioral tests is the laboratory environment, especially for the open-field and plus maze tests^57,58^.

The distinct phenotype observed between young and adult *Scn2a*^+/−^ mice suggest that the abnormal behaviors observed in young mutant mice can be rescued to some extent in adulthood by compensatory mechanisms that might emerge at the end of the juvenile/adolescent period of development (after P44). Notably, this compensation should not be directly linked to the physiological switch between Nav1.2 and Nav1.6, because it occurs at around P20 in mouse brain^13^, and could instead be linked to other more complex homeostatic mechanisms triggered by Nav1.2 haploinsufficiency. Indeed, when activity is perturbed in neuronal networks, different homeostatic mechanisms that attempt to restore the initial condition can take place^59^. Decreased expression of Na_v_1.2 channels may result in alterations in the excitatory/inhibitory (E/I) balance, a central concept in ASD etiology^60,61^. Nelson and Valakh^62^ have proposed that homeostatic mechanisms which take place in ASD during development in response to E/I imbalance in some circuits may be insufficient and can become maladaptive amplifying the imbalance in other circuits. In *Scn2a*^+/−^ mice, compensatory mechanisms might be relatively efficient to restore behavioral dysfunctions at adulthood. Of note, compensatory processes in ASD patients during development are thought to underlie the reduced severity of autistic symptoms reported in some individuals at adulthood^63^. Alternatively, adult *Scn2a*^+/−^ mice may display a mixed phenotype relevant to both autism and schizophrenia, as proposed by Tatsukawa *et al*.^26^. Indeed, both disorders share some clinical manifestations, such as deficits in social communication, but schizophrenia typically emerges in late adolescence or early adulthood. Moreover, schizophrenia and autism can overlap in some adult patients that tend to display an atypical phenotype^64–67^, and, more importantly, several studies have reported loss-of-function SCN2A mutations/variants in schizophrenic patients^10,38,68^. Unfortunately, no detailed clinical information is available about these patients.

In summary, our data demonstrate that Na_V_1.2 haploinsufficiency leads, in young male mice, to social, communicative, motor, cognitive and emotional dysfunctions that are relevant to ASD and comorbid phenotypes. By contrast, adult mice show only communicative deficits. There are similarities between the phenotype of young *Scn2a*^+/−^ mice and that of some young autistic patients with *SCN2A* mutations, indicating that these mice can be an ASD model with construct and face validity during the juvenile/adolescent period of development. However, it is not possible to correlate the phenotype of adult *Scn2a*^+/−^ mice with that of adult ASD patients that carry *SCN2A* mutations because, in our knowledge, information about clinical features of these patients is not available. Further investigations in *Scn2a*^+/−^ knockout mice at the cellular and circuit level may provide important new insights into mechanisms contributing to these phenotypes and possible homeostatic responses that can attenuate their severity.

## Materials and Methods

### Animals

Generation and genotyping of *Scn2a*^+/−^ and *Scn2a*^−/−^ mice have been described by Planells-Cases *et al*.^69^. Original heterozygous breeders were provided by Dr. E. Glasscok of Louisiana State University Health Sciences Center, Shreveport^70^. The heterozygous *Scn2a*^+/−^ and control *Scn2a*^+/+^ littermate mice that we used in the experiments were generated from crosses between heterozygous *Scn2a*^+/−^ males and wild-type females on a C57Bl6/J background (Charles Rivers, France). All mice were housed in a controlled environment (22–24°**C**, 40–50% humidity) under a 12 h dark/light cycle with access to food and water *ad libitum*. After weaning (post-natal days 21–23), mice were group- housed with same sex littermates of mixed genotype (n = 3–5/cage) in standard polycarbonate cages containing plastic houses and nesting material. Only male mice were used for the behavioral characterization of *Scn2a* knock-out mice, since ASD affects males more frequently than females. Behavioral tests were performed sequentially in young (between P22 and P44, corresponding to the juvenile (P21–28) and adolescent (P29–45) periods^71^) and adult (between P60 and P95) male mice. For all experiments, the animals were habituated to the testing room at least 30 min before the beginning of tests. Heterozygous *Scn2a*^+/−^ mice (n = 14) and their control littermates (n = 17) (from a total of 7 litters) were submitted to the behavioral tasks in the following order: anxiety tests, reciprocal social interaction, marble burying test, ultrasonic vocalizations, memory tests, rotarod and tail suspension test. All the tests were conducted in young and adult mice except one memory task, the Barnes maze test, which was not evaluated in young mice in order to perform all the behavioral experiments before the end of the adolescent period. An additional group of male mice (from a total of 5 litters) was used to assess olfactory function in young then adult mice (n = 14 for each genotype). All procedures were performed in accordance with the European Council Directive (2010/63/EU) and approved by the local ethical committee (Comité Institutionnel d‘Éthique Pour l’Animal de Laboratoire (CIEPAL) -Azur, https://ciepal-azur.unice.fr/) and the French Ministry of Research (http://www.enseignementsup-recherche.gouv.fr/, APAFIS#11438-2017090817345631).

### Ultrasonic vocalizations (USV)

To investigate mice social communication, USV were recorded in a sound-attenuating chamber using an ultrasound microphone (CM16/CMPA, Avisoft bioacoustics, Glienicke, Germany) positioned above the cage. In juvenile mice, pairs of same age, sex and genotype from different litters were simultaneously introduced into the cage after 1 h of social isolation and vocalizations were recorded for 5 min^72^. In adult mice, courtship USV were emitted by male after placing a C57BL6/J estrus female in the recording chamber during 3 min^73^. USV spectrograms were generated using Avisoft SASLab Pro software (Avisoft bioacoustics). The number, duration and peak frequency of ultrasonic calls were analyzed.

### Olfaction test

Since olfactory impairment could interfere with the social behaviors measured in male mice, olfactory discrimination between social and non-social scents was assessed as previously described with minor modifications^74^. Squares of filter paper (5 × 5 cm) were soaked with water or coffee (non-social scent) (approximately 0.5 ml of solution). Social odors were generated after placing filter paper squares during 3 days in cages housing non familiar juvenile males or adult females and used to test young or adult male mice, respectively. Mice were placed in clean plexiglas cages without bedding for 10 min of habituation. Then, scents were introduced sequentially (water, coffee, social odor) for 3 min each, spaced by 1 min interval. Olfactory preference corresponds to the difference between the time spent sniffing social or non-social scents and that spent sniffing water. Social odors are attractive whereas coffee is aversive.

### Reciprocal social interaction test and self-grooming behavior

Social interaction was evaluated in freely moving pairs of mice as previously described with slight modifications^75^. The test mouse was introduced in the open-field arena and allowed to explore for 20 min. The time spent in spontaneous repetitive grooming behavior was measured during the last 10 min. Then, an unfamiliar, age-matched, C57Bl6/J male wild-type stimulus mouse was placed in the arena and the behavior of the pair of mice was videotaped for 5 (adult) or 10 (juvenile) min. Total time spent in social interactions (such as nose-to-nose or anogenital sniffing, following or mounting) initiated by the test mouse toward the stimulus mouse was measured manually by the same experimenter, who was blind to the genotype of test mice. The latency for the first social contact was also analyzed.

### Marble burying task

Repetitive behavior related to digging was assessed using the marble burying test as described by Thomas *et al*.^28^. Juvenile or adult mice were placed into a transparent Plexiglas box (30 × 30 × 30 cm) or a polycarbonate cage (57 × 20 × 40 cm) respectively, containing a 5 cm layer of fresh bedding (poplar wood chips, average particle size 3.5–4.5 mm; Lignocel Select, SAFE, France). Twenty marbles were disposed in five spaced rows of four marbles on the bedding surface. The number of marbles with more than 50% of their surface covered were scored as buried during a 20 min session. The number of buried marbles was continuously scored during the test by the same investigator blind to the animal genotype.

### Rotarod

Motor coordination, balance and motor learning were assessed using an accelerating rotarod (Bioseb-*In vivo* Research Instruments,Vitrolles, France). Mice were habituated to the procedure before the test phase: each mouse trained until they were able to stay 1 min on the circular rod at a constant speed of 4 rpm. Mice were then placed on the rod that accelerated from 4 to 40 rpm over 5 min. Mice were given three trials a day for 2 consecutive days with an inter-trial interval of 30 min^49^. Latency to fall from the rotating rod was automatically recorded.

### Anxiety tests: Open-field and Elevated-plus maze tests

The open-field test is commonly used to assess exploratory behavior and anxiety in rodents. The white and opaque arena (40 × 40 × 30 cm) was brightly illuminated at 300 lux. Mice were allowed to explore the arena for 30 min. The distance traveled and the time spent in the periphery (6 cm from the walls) and the center (20 × 20 cm) of the open-field as well as the total distance in the entire arena were automatically measured using ANY-maze video-tracking system (Stoelting Europe, Dublin, Ireland).

Anxiety behavior was also evaluated using the elevated-plus maze test. The maze, slightly illuminated (30 lux), consisted of two open arms (25 × 5 × 2.5 cm) and two enclosed arms (25 × 5 × 25 cm) elevated 40 cm above the floor. Mice were placed in the center of the maze and allowed to explore the maze for 5 min. An arm entry was defined as all 4 paws of the mouse in an arm. The number of entries and the time spent in the open and the closed arms was determined using ANY-maze video-tracking system.

### Tail suspension test (TST)

Responsiveness to an inescapable stressful situation was measured using the TST test. Mice were suspended by the tail to a hook using adhesive tape. The time spent immobile, thought to reflect a resignation state, was recorded during a 6-min session through an automated device (Bioseb). Mice which climbed their tail were removed from analysis.

### Spontaneous alternation behavior

Spatial working memory was assessed in a symmetrical grey plastic Y maze (30 × 6 × 15 cm). Mice were placed at the end of one arm and allowed to freely explore the maze for 8 min. The sequence and the number of arm entries were manually recorded. An alternation was defined as consecutive entries into the three different arms (A, B or C) on overlapping triplet sets (ex: ABCABBACAB = 5). The alternation score (%) was calculated as the ratio of actual (total alternations) to possible (total arm entries-2) alternations x100.

### Novel object r**e**cognition task

This memory test based on the spontaneous tendency of rodents to explore novelty was performed as previously described^76^ in an open-field apparatus illuminated at 30 lux. During the phase of habituation (Day 1), mice were allowed to freely explore the arena during 5 min and then to explore two identical objects for 10 min. On experimental day (Day 2), mice were submitted to two trials with an intertrial interval of 1 hour. During the first trial (acquisition phase), mice were placed in the open-field in the presence of two identical objects (different from Day 1) and stayed the time necessary to explore for 20 s the two objects. Mice that did not explore the objects within a 12 min period were excluded. During the second trial (retention test), one of the objects was replaced by a novel object and mice were allowed to explore both objects (novel and familiar) for 5 min. Mice that did not explore the objects for a minimum of 15 s (young) or 20 s (adult) were excluded. The pairs of objects used were different in shape, size (height between 12 and 19 cm) and texture (glass, plastic or metal). The two objects were placed symmetrically in the center of the arena about 6 cm away from the wall. The location of the novel object was counterbalanced across animals (right or left side). Recognition memory was evaluated using a discrimination index (DI). This index corresponds to the difference between the time spent exploring the novel object and the familiar object divided by the total time of exploration of both objects and is expressed in percentage.

### Barnes maze test

Spatial learning and memory were tested using the Barnes maze. The maze consisted of a white circular platform (90 cm diameter) with 20 holes (5 cm diameter) along the perimeter. Mice can access to an escape box hidden under one of the hole (target hole). Extra-maze visual cues were placed on the walls of the testing room in order to help the mice to learn the position of the target hole. During the phase of acquisition (day1–4), mice received three trials per day separated by 30 min. At the beginning of each trial, mice were placed in the center of the maze under a rectangular opaque box. The box was removed 10 s after the onset of light (700 lux) and mice were allowed to explore the maze. The trial ended when mice entered the escape box with all four paws or after 3 min have elapsed. In the latter case, mice were gently guided to the escape box by the experimenter and remained there for 1 min. For each trial, the latency to enter the escape box was measured. The day after the acquisition phase (day 5) and one week after (day 12), mice were submitted to a probe trial for 90 s without the escape box. The latency and the distance traveled before finding the target hole as well as the number of visits to each hole were automatically recorded using ANY-maze video-tracking system. The Barnes maze were divided into 4 quadrants: the correct (where the target hole was located), opposite, left and right quadrants. Measurements of time spent in each quadrant were also made on days 5 and 12.

### Statistical analysis

Data were analyzed for normality (Shapiro-Wilk test) and equal variances using Origin 8.0 (OriginLab Corporation, Northampton, USA). When data were normally distributed, comparisons between genotypes in young or adult mice were evaluated using unpaired Student’s t-test or Welch’s t-test (when variances were different), otherwise non parametric tests were applied. When appropriate, a two-way repeated measures ANOVA followed by Bonferroni’s post-hoc test or paired t-test (for within subjects comparisons) was used. One sample t-tests were used to compare the discrimination index with a 0% chance level in the novel object recognition test and the time spent in the target quadrant to a 25% chance level in the Barnes maze test. The results are expressed as mean ± S.E.M. We considered the differences as statistically significant with p ≤ 0.05. * indicates significant differences in comparisons between genotypes and # or letters indicate significant differences in comparisons within subjects or with chance level. We considered borderline significance when p-values were between 0.05 and 0.09.
